# Attitude theory and measurement in implementation science: a secondary review of empirical studies and opportunities for advancement

**DOI:** 10.1186/s13012-021-01153-9

**Published:** 2021-09-14

**Authors:** Jessica Fishman, Catherine Yang, David Mandell

**Affiliations:** Perelman School of Medicine, University of Pennsylvania, 3535 Market Street, Floor 3, Philadelphia, PA 19104 USA

**Keywords:** Psychology methods, Validated measurement, Instrumentation, Causal theory, Predictive models

## Abstract

**Background:**

Implementation science studies often express interest in “attitudes,” a term borrowed from psychology. In psychology, attitude research has an established methodological and theoretical base, which we briefly summarize here. We then review implementation studies designed to measure attitudes and compare their definitions and methods with those from psychology.

**Methods:**

A recent review identified 46 studies empirically examining factors associated with implementation. For each of these studies, we evaluated whether authors included attitudes as a construct of interest, and if so, whether and how the construct was defined, measured, and analyzed.

**Results:**

Most of the articles (29/46 [63%]) mention attitudes as an implementation factor. Six articles include a definition of the construct. Nineteen studies were designed to measure attitudes but lacked clarity in describing how attitudes were measured. Those that explained their measurement approach used methods that differed from one another and from validated methods in social psychology. Few articles described associated analyses or provided results specific to attitudes. Despite the lack of specificity regarding relevant measurement, analysis, and results, the articles often included causal conclusions about the role of attitudes.

**Conclusions:**

Attitudes may be an important construct to implementation scientists, but studies to date are ambiguous in their definitions of attitudes and inconsistent in the methods used to measure and analyze attitudes. We discuss how implementation studies can apply psychology’s standardized definitions, validated measurement approaches, and causal models that include attitudes. This application of attitude theory and methods could offer implementation research valuable scientific opportunities.

Contributions to the literature

- Implementation science often posits that attitudes are an important factor influencing implementation. Yet, few studies apply standardized practices from attitude theory or measurement.
- We document conceptual ambiguity and use of methods that are inconsistent and also discuss how advances in attitude measurement and theory from social psychology can help implementation science (1) establish a common approach with shared language, (2) pool data from multiple studies, (3) estimate the predictive validity of attitudes, and (4) identify when effective implementation strategies are likely to be those that target attitudes.

## Background

Within every scientific discipline, scientists strive to develop both uniform definitions and measurement methods for important constructs. Shared terminology and standardized methods enable scientists to collectively advance relevant theory by testing relationships among variables of interest [1]. Implementation science has been described as “somewhat elusive and overwhelming for researchers” because it has not yet developed distinct construct definitions and associated psychometrically sound instrumentation [2]. These challenges can be observed in the use of the term “attitudes” and its associated measurement in implementation studies. Implementation scientists’ interest in attitudes is evident from some of their earliest and most prominent measures and frameworks. However, it is not clear whether implementation scientists are using a common definition of attitudes or standardized measurement. It is also unclear whether their definitions and measurement are consistent with validated approaches commonly used in social psychology.

Implementation science is a relatively new field that seeks to build on many existing disciplines, especially organizational, industrial, and social psychology. Attitude research has historically been a product of psychological research, but is also relevant to implementation science’s explorations of how cognition can influence behavior in a professional context — specifically, whether individuals in an organization use evidence-based practices [1, 3, 4]. In this review, we provide a brief three-part summary of psychology’s advances in attitude research that may be relevant to implementation science. We then review recent implementation studies to examine their consistency with one another and psychological research. Finally, we offer suggestions for implementation scientists who wish to measure attitudes towards the use of evidence-based practices in their research.

### Defining attitudes

The psychologist Herbert Spencer is credited with first using the term “attitude” in 1862 [5]. In the early twentieth century, Gordon Allport declared that the concept of attitude is “probably the most distinctive and indispensable concept” in psychology [6]. Throughout much of the twentieth century, psychologists debated its meaning; the debates waned only in the last few decades of that century [7, 8]. Previously, a wide variety of concepts were labeled “attitude” [9, 10]. The lack of distinction limited the discipline’s ability to understand attitude in terms of its relationships to other constructs [11].

In the 1920s, Louis Thurstone and others argued that the distinctive feature of attitude was an evaluative or affective predisposition towards an object, idea, or issue [12]. Thurstone is credited with developing a formal technique for examining attitudes. To indicate one’s attitude towards an issue, he paired statements with numerically scaled response options so he could calculate the degree to which one judged that issue favorably or unfavorably. In line with Thurstone, major mid-century social psychologists like Martin Fishbein and Icek Ajzen conceptualized an attitude towards a behavior as an evaluative response that predisposed one favorably or unfavorably towards performing that behavior [9]. For example, if one believes that performing a behavior has mostly positive consequences, then one’s attitude would be supportive (or in favor) of performing that behavior. In contemporary psychology, one’s attitude towards a behavior is generally still defined as the degree to which one has a positive versus a negative evaluation of performing the behavior [8, 10, 13].

Applied to implementation research, an attitude would be conceived as how favorably or positively one is predisposed towards using a particular evidence-based practice (EBP). This predisposition is established by one’s beliefs about the consequences or outcomes of using that EBP, which may be viewed as an advantage or disadvantage of taking that action. For example, practitioners’ attitudes towards using an EBP may be based on whether they believe that using the EBP will be relatively pleasant versus unpleasant, necessary versus unnecessary, beneficial versus harmful, or simply good versus bad [14].

### Attitudes in causal models

Throughout much of the twentieth century, psychologists debated whether attitudes could predict behavior [9, 15–17]. Early results were disappointing [9]. However, advances in psychology have led to the general consensus that attitudes towards a behavior can predict behavior by way of a meditator: behavioral intention, which is the strongest predictor of behavior [18, 19]. Fishbein and Ajzen pioneered the study of behavioral intention, describing it as the volitional and immediate antecedent of behavior. The construct is used to represent the subjective probability that one will perform a given behavior, as well as the amount of effort one is likely to exert [19, 20]. In implementation research, the intention to use an EBP has been shown to predict its use [1, 21].

According to several causal models, behavioral intention is a function of attitudes and other psychological variables, such as subjective norms and self-efficacy [22]. Experimental and observational data support these relationships, and the proposed causal pathways predict behavior [23]. However, some types of attitudes are much more important in predicting behavior than others.

Attitudes toward an object, person, policy, or concept are unlikely to predict behavior [13, 23]. For example, attitudes toward doctors or health care policy or even health and disease will not predict whether one receives flu vaccinations, cancer screenings, or other evidence-based medical interventions [13, 23]. When the goal is to predict a behavior (including use of an EBP), it is important to study attitudes towards the specific behavior of interest, which may have a decisive influence on one’s strength of intention to perform that behavior and, in turn, one’s actual performance of that behavior [9, 19, 23, 24]. As discussed below, this theoretical advance is reflected in standardized measurement approaches.

### Advances in attitude measurement

Throughout the twentieth century, the field of psychology explored hundreds of different quantitative measurement procedures. Oftentimes a procedure was unique to one study and lacked justification other than the investigator’s intuition, leading to “conflicting results and different conclusions concerning the relations between attitude and other variables” [25].

Psychology has since standardized methods for measuring attitudes, namely the Thurstone and Likert attitude scales [9, 10, 26]. Psychologists typically measure the degree to which one has a positive versus a negative evaluation of the behavior using a set of bipolar semantic differential scales [8, 25–27]. Bipolar adjectives may include pleasant-unpleasant, wise-foolish, beneficial-harmful, necessary-unnecessary, useful-useless, and, more simply, good-bad [25–27]. To measure therapists’ attitudes towards using a particular EBP at each patient session, the therapist could rate how beneficial versus harmful and pleasant versus unpleasant it would be for them to do so [3]. These adjectives usually anchor a 5- or 7-point scale. The responses are aggregated and used to assign a respondent a single number representing how favorably or unfavorably the individual regards a behavior. There are several detailed guidelines on scale construction and analyses [14, 19, 27, 28].

Psychologists have also developed validated, qualitative methods of assessing the beliefs that underlie attitudes. Standardized belief elicitation studies ask individuals what they believe to be the advantages and disadvantages of performing a behavior [29, 30]. This literature also developed validated procedures for analyzing qualitative data and using it to tailor quantitative assessments of attitudes, based on the population of interest. Applied to implementation research, investigators conducting a belief elicitation would ask a practitioner to share what they believe to be an advantage or disadvantage (or good or bad) about using a particular EBP in the recommended context [31, 32].

When assessing attitudes qualitatively or quantitatively, if the ultimate goal is to predict behavior, it is important to measure attitudes towards that specific behavior of interest rather than a general group of behaviors, a concept, policy, person, or object [25–27]. This measurement advance is referred to as the principle of correspondence; Presseau et al. have recently highlighted its applicability to implementation science [24]. Fishbein demonstrated that when behavior is defined as taking a certain action in a certain context, attitude items should refer to the same action in the same context. Attitudes will vary depending on the behavior specified [24]. For example, when studying therapists’ implementation of CBT, therapists may have different attitudes depending on the CBT component that is of interest and the clinical context for using that CBT component [3]. For example, therapists’ attitudes towards using exposure therapy with children can differ from their attitudes towards using agenda setting or giving homework for the same population. Therapists’ attitudes towards using exposure therapy with children may also differ from their attitudes towards using exposure therapy with adults.

To our knowledge, this study represents the first attempt to compare attitude measurement and theory not only among implementation studies but also with definitions and methods from the field of psychology. Our goal was to examine the degree to which approaches can vary, because variation reveals opportunities to adopt shared definitions and apply validated, standardized methods. This review is part of a broader ongoing effort to increase the use of validated methods and theory among implementation studies. We therefore build on the work of others who have highlighted many ways that social psychology has contributed and can further contribute to implementation science [33, 34].

## Methods

### Identifying articles for review

In 2020, Lewis et al. published a review of recent implementation studies that included empirical assessments of mediators, moderators, and mechanisms [35]. Their search identified 46 published studies. For three reasons, we selected these 46 studies for our analyses. First, this review was published in the field’s flagship journal, suggesting that it was judged to be a rigorous and comprehensive review. Second, because implementation science has been described as a rapidly evolving field, we sampled only articles published relatively recently [33, 34]. Older articles may not reflect pertinent advances made in implementation science. For example, Godin et al. [36] identify several studies that reference attitudes; however, these are much older publications, which may not represent current practices in implementation science. Presumably, the more recent articles included by Lewis et al. were able to benefit from relevant earlier research and represent the field’s current practices.

Third, given that a chief objective of ours was to examine how attitudes are measured, an ideal sample is one in which articles are likely to include empirical assessments of attitudes. In psychology, attitudes are examined as mediators, and all the articles in our sample include some empirical assessment of a mediator, moderator, or mechanism. Other types of articles could mention attitudes but might be much less likely to measure attitudes empirically. To illustrate, a review by McIntyre et al. suggests that empirical analyses of this kind are very rare [37]. They reported that just seven out of 123 articles conducted empirical assessments of a theory-based construct (such as attitudes). We reviewed these seven studies and found that three did not study attitudes.

To qualify studies for inclusion in their review, Lewis et al. searched the PubMed and CINAHL Plus databases specifically for studies published in English that qualified as empirical implementation studies and involved the quantitative or qualitative exploration of mechanisms, mediators, or moderators. Lewis et al. systematically identified such articles that were published between January 1990 and August 2018. For the reasons noted above, this sample was not designed to be representative of the wide-ranging collection of existing implementation research.

### Data collection

For each article in our sample, two coders extracted data. Inter-rater reliability was high for extraction of each type of data and coding (with coefficients of .90 or greater for **Krippendorff’s alpha or** Cohen’s kappa, and average percentage agreement of 94%, where appropriate); any discrepancies were resolved by consensus among authors. First, coders determined if the term “attitude” was used at least once in the text of the article. Among those that did, the coders reviewed if and how the authors defined and operationalized the term. Coders also tracked whether the authors assessed attitudes qualitatively or quantitatively, and how they measured attitudes, including whether attitudes were measured towards a behavior, such as the use of a particular EBP. The coders also recorded whether a study examined attitudes exclusively or in conjunction with other variables. Lastly, coders determined if the authors explained why they were studying attitudes (as opposed to other constructs). For this purpose, we tracked whether a validated model of behavior prediction informed their study of attitude, or if they otherwise justified their interest in this variable.

## Results

### Defining “attitudes”

Of the 46 empirical implementation studies reviewed, 29 [63%] include the term “attitude.” Among these 29 articles, 6 include a definition (or some conceptual description how the construct can be operationalized), as shown in Table 1. These definitions were similar across articles, and to the definition commonly used in psychology. For example, Garner et al. [38] defined attitudes toward using an evidence-based practice as the “positive or negative evaluations of behavior.” Bonetti et al. [39] stated: “Attitudes towards the behaviour are proposed to arise from a combination of beliefs about its consequences (behavioural beliefs) and evaluations of those consequences (outcome evaluations).” Carrera and Lambooij [40] define attitudes as “the sum of (positive and negative) beliefs weighted by evaluations of those beliefs.” Each of these descriptions reflects the seminal attitude research of social psychologists Martin Fishbein and Icek Ajzen.

**Table 1:**
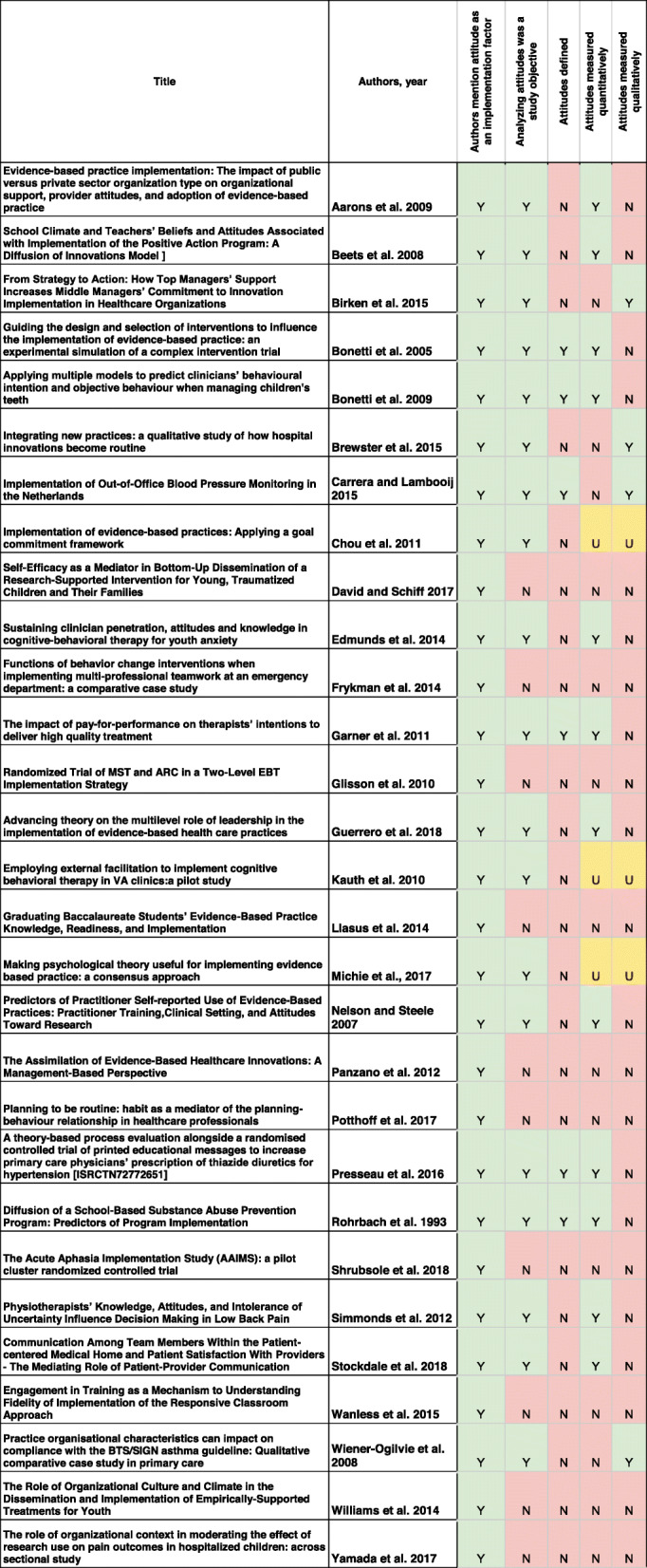
The sample of articles and their characteristics

Among the other 23 articles, some authors implied that attitudes are related to “barriers” or “facilitators” of implementation. For example, Brewster et al. [41] present their interest in learning about “facilitators” and consider “attitudes” as a “facilitator” but not a “barrier.” Other studies seem to consider attitudes to be conceptually distinct from “barriers” and “facilitators” [39, 42, 43]. Yet others study “barriers” and “facilitators” to implementation without clarifying whether either term is related to their examination of attitudes.

A study by Michie et al. [43] sought to identify theoretical constructs important to EBP implementation and “simplify [them] into construct domains.” In a table, they identify one of the domains as both “beliefs about consequences” and “anticipated outcomes/attitudes.” For this domain, they list 13 “component” constructs that include “attitudes” and “beliefs.” Within this domain, the other component constructs are wide ranging: “punishment,” “incentives/rewards,” “sanctions/rewards,” “salient events,” “critical incidents,” “unrealistic optimism,” “contingencies,” and “threat.” The domain and its component terms are not defined. A related publication by these authors [44] proposes a refined “Theoretical Domains Framework” by presenting 14 components as the ideal number of theoretical domains and revises some of the labels.

Investigators that aimed to examine attitudes also report assessing other potentially related concepts, such as perceptions of “relevance” [38, 43], “usefulness” [44–48], “acceptance” [44], and “appropriateness” [39, 40, 45]. In each case, it is unclear if the authors define or operationalize these terms differently from attitudes. For example, given that Carrera and Lambooij [40] and Bonetti et al. [39] offer a definition of attitudes that does not refer to “relevance” or “usefulness,” it could be assumed that the authors treat them as distinct concepts.

### Data used to study attitudes

Among the 29 articles that mention attitude as an implementation factor, 10 were not designed to measure attitudes but do discuss attitudes when reviewing prior studies*.* For example, based on the implementation science literature, Williams et al. [46] conclude that “organizational culture has been empirically linked to clinician attitudes.” The remaining 19 articles reported on studies designed to analyze attitude data, but three of these articles do not indicate whether they used qualitative or quantitative data (Chou et al. [47]; Kauth et al. [48]; Michie et al. [43]). The 16 articles that explain their qualitative or quantitative data collection are described in the sections below.

#### Quantitative data

Twelve articles report that they used a quantitative measure of attitudes. Three of these studies [46, 49, 50] used a 15-item instrument, the Evidence-Based Practice Attitude Scale (EBPAS). The EBPAS developers acknowledge that the instrument assesses other constructs, such as knowledge [51]. For example, three items measure knowledge of “requirements to adopt new practices,” including state regulations. It also is worth noting that the EBPAS items refer only to a vaguely defined behavioral goal, such as adopting “new practices.”

Nine studies used other instruments that reflect different conceptual approaches from the EBPAS and from one another. For example, Stockdale et al. [50] examined attitudes using the Emotional Exhaustion subscale of the Maslach Burnout Inventory, which asks participants to “indicate how frequently you experience each feeling or attitude” referenced by nine statements, including “I feel I’m at the end of my rope” and “I feel I’m working too hard on my job.” Edmunds et al. [51] report using another study’s unpublished “Clinician Demographics and Attitudes Questionnaire” in addition to the EBPAS, and it is not clear how these two approaches to measuring attitudes were reconciled. Beets et al. [52] adapted items based on Dane and Schneider’s [53] “assessment of quality of delivery.”

Other studies report using part of an existing instrument but do not describe which ones and whether they applied some kind of summary index. Some authors report measuring attitudes using their own quantitative approach. For example, Rohrbach et al. [45] described hypothetical scenarios about a character named Joe and asked, “Would most of your friends think what Joe did was OK?” Those authors used the dichotomous yes/no answers, along with other responses, to create a numeric scale.

A few measures resemble validated ones from social psychology [14]. Bonetti et al. [39] describe the methods used to empirically assess the beliefs that may underlie attitudes towards referring patients for lumbar X-rays; they also include some questionnaire items. For example, they state: “Three items that assessed behavioural beliefs that referring for a lumbar X-ray would result in a particular consequence (‘reassure the patient’, ‘allay my uncertainty’, ‘make me more confident about managing the patient’s symptoms’) were rated on a 7-point scale…” They also explain that they summed the scores, with higher values representing stronger attitudes in support of referring patients for lumbar X-rays.

#### Qualitative data

Four articles used qualitative methods to examine attitudes [38, 39, 44, 54]. None clearly describe which data were considered relevant to attitudes or how the data were analyzed. There were no indications as to whether attitudes towards a behavior (i.e., the use of a certain EBP) were examined. The authors did make conclusions about attitudes that were attributed to empirical analyses. For example, Brewster et al. [41] argued that attitudes “changed over time” but did not explain which data were categorized as attitudinal or how attitude change was analyzed.

As another example, Carrera and Lambooij [40] state that they studied attitudes of physicians and patients, but it is not clear if questions were asked to identify attitudes towards performing a particular behavior. For the analyses, transcripts from focus groups were “analyzed by qualitative content analysis,” yet it is not apparent which physician or patient data were content analyzed as attitudes. The authors report: “We found that physician’s attitude enabled the use” of one practice “while it impeded the use of” another. They add that, among patients, the direction of the effect was the opposite, where implementation was said to have “affected their attitude.” The authors did not explain how qualitative data were analyzed to make these causal determinations. It is also unclear if Carrera and Lambooij consider the data on “acceptance” to be measuring a construct distinct from attitudes. Tables 2, 4, and 5 list “attitudes,” “perceived usefulness,” and “acceptance” as separate factors; however, these constructs are conceptually merged in the manuscript’s main text and it is not clear if these terms actually pertain to the same data.

### Attitudes in relation to other variables

All 29 articles describe studies that were designed to examine attitudes and other constructs. Some authors refer to validated models of behavior prediction that specify relationships between attitudes and other constructs. For example, Bonetti et al. [39] and Presseau et al. [55] refer to the Theory of Planned Behavior, which represents the role of attitudes within a network of other psychological constructs connected by causal pathways. In line with this model, Bonetti et al. [28] note that behavioral intention is a function of attitudes, subjective norms, and perceived behavior control. The Theory of Planned Behavior was the most commonly mentioned causal model [38–40, 42, 43, 46, 52, 56, 57]. However, descriptions of this model were often missing or not consistent with how the developers of this model describe it.

Some studies described roles that attitudes might play that conflict with existing evidence. For example, Edmunds et al. [51] describe the “usefulness of EBPs” as being determined by attitudes towards EBP. Conversely, Carrera and Lambooij [40] write that attitudes are the result of beliefs that “are influenced by perceived usefulness, which is the degree to which an individual believes that usage would be beneficial, and perceived ease of use, or the degree to which an individual is convinced that usage would not be arduous.” This is contradicted by their “analytic framework” description, however, in which attitude is “influenced by social norm, or the impact of one’s social environment, and enabling conditions, which are objective factors in the environment that promote action.” Some authors propose that attitudes should be studied because they function as an “integrating mechanism” [41], a source of bias [58], or a “facilitator of acceptance” [40]. Most authors did not mention if or how attitudes may relate conceptually to the other constructs of interest.

## Discussion

We found that oftentimes when investigators study attitudes, they do not define this construct. As Martinez et al. observe, implementation science could benefit from “carefully defining” a construct of interest, “ideally based on existing theory or available definitions” [2]. We recommend adopting the standardized definition of an attitude that is widely used in social psychology. An explicit definition of attitude can inform procedures for measuring this construct [25]. This standard definition would distinguish attitudes from other constructs with which they often are conflated, including willingness, intention, and self-efficacy. Definitions would also help clarify whether investigators intend to distinguish attitudes from other terms, such as “acceptability,” “appropriateness,” and “barriers and facilitators.” Without clear definitions, there are many opportunities for investigators to lack agreement on the meaning of constructs. For example, when developing a “Theoretical Domains Framework,” participants who “possessed a good understanding of behaviour change theory” were asked to interpret the meaning of attitudes and various other terms that were not defined. They found different ways to interpret the meaning of these terms and their theoretical relationship to each other [44].

In the studies explored in the present review, authors rarely explain how attitudes were measured. When they do, their accounts suggest that attitudes were measured in unrelated ways among different studies. Research in social psychology has shown that, with fundamentally different approaches to measuring attitudes, “a study can lead to apparently conflicting results and different conclusions concerning the relations between attitudes and other variables” [25]. Implementation science has the opportunity to apply validated, standardized measures of attitudes with guidance tailored specifically to implementation researchers [14].

In particular, future studies can measure attitudes towards using a particular EBP in a specified context and then test the degree to which attitudes explain variance in a population’s strength of intention to perform that behavior [59]. As documented by a systematic review of implementation science, behaviors are rarely specified clearly [60]. In turn, Presseau et al. warned, “Despite half a century of guidance on behaviour specification, research is frequently published in which the behaviour is poorly specified” [13]. Presseau et al. have argued that implementation science could improve the measurement of theoretical constructs by specifying the behavior of interest. This guidance can be applied specifically to attitude measurement. The present review found that studies measured attitudes towards general categories, such as “new practices” or “evidence-based practices” rather than a specific behavior.

The conceptual and methodological problems documented by this review are similar to those that the field of psychology faced in the early part of the twentieth century, as encapsulated by the following statement by Fishbein and Ajzen: “In addition to a lack of agreement on the definition of attitude, different noncorrelated operations can be found for the same concept, and the identical operation is often given different conceptual labels” [15]. They added that, as a result of this conceptual and methodological neglect, attitude research has largely been “noncumulative and has failed to produce a systematically integrated body of knowledge.” Without standardized approaches, it is difficult for the research to develop a common scientific language, compare or pool findings across studies, or develop theories that can establish causal mechanisms of implementation [2, 54].

### The utility of causal models

There is a great deal of empirical evidence about the role of attitudes based on psychological studies of many different behaviors. The lessons learned from psychological research about behavioral prediction and change are directly applicable to implementation research. Implementation research examines behavior within organizations. The discipline of psychology includes organizational psychology, which has the same goal [61, 62]. Both fields are concerned with identifying the determinants of behavior. When citing Eccles et al. [63], Presseau et al. [55] summarize: “Behavioural science has systematically operationalized theories concerning determinants of behaviour and how they are associated with each other. This may be useful for understanding the mechanisms underlying implementation interventions designed to change clinicians’ behaviour.” Godin et al. [36] also observe: “The problem of understanding why healthcare professionals do or do not implement research findings can be viewed as similar to finding out why people in general do or do not adopt a given behaviour such as health-related habits.” They stress, “This has been extensively investigated, and social psychological theories have already demonstrated their value.” In contrast to causal models, “frameworks” (such as the Theoretical Domains Frameworks) rely on intuition to identify the domains and constructs that seem to be “the most suitable.” Such frameworks do not identify which domains or constructs have causal relationships with one another. Well-tested models, on the other hand, represent the results of empirical tests (spanning many decades) that demonstrate the predictive validity of specific constructs and their causal pathways, which allow studies to identify the mechanisms of behavior change and design interventions to target them.

Theories that have demonstrated predictive validity, such as the Theory of Planned Behavior [64], Unified Theory of Behavior [65], and the Integrated Model [66], are based on evidence that attitudes can influence behavioral intention. These theories also posit that, in addition to attitudes, perceived behavioral control (or self-efficacy) and subjective norms can influence behavioral intention. This proposition could be tested within implementation research to better understand the degree to which attitudes explain variance in a study population’s motivation to implement a particular EBP. Implementation science can also test which variables influence attitudes. By designing studies to evaluate theorized relationships between constructs in causal models, the results can inform the development of implementation strategies. Implementation strategies are likely to be effective and efficient if they target the malleable constructs that predict outcomes [1].

When testing a causal model, it is important to empirically establish the degree to which attitudes contribute, since the role attitudes play will vary depending on the population and specific behavior of interest. When attitudes explain a substantial proportion of variance, intervention strategies can be developed to change attitudes — which are malleable — and potentially increase EBP uptake. For example, in many studies, attitudes drive intention to a greater extent than do subjective norms [21]. In other cases, subjective norms are more influential than attitudes [13, 67, 68]. Depending on which variables are predicting intention to use an EBP, implementation strategies can be designed to target the most influential determinant of intention, and strategies that do not may be less effective and efficient [67, 68].

When considering the limitations of the current study, it is important to note that the articles reviewed may not be representative of other implementation studies. Indeed, we purposefully selected a sample of articles that empirically studied mediators and moderators. As documented by McIntyre, implementation science has a high proportion of articles that express interest in studying theory-based constructs, such as attitudes, but forgo an empirical assessment. In a secondary analyses of the articles included in McIntyre’s review, we found that 4% (4/123) provided information on attitude measurement, and doing so was more common in our primary sample. Given the breadth of research in implementation science, this review is not intended to represent the wide-ranging variety of studies that could be sampled.

In addition, the present review was limited to recent articles [33, 34]. Given that our results are based on recent publications, the results may be less relevant to older implementation research. For example, a review by Godin et al. [36] identifies several articles that mention attitudes but these articles are not included in our sample. Future research could investigate older studies in implementation science to determine if attitude theory and measurement has improved over time.

## Conclusion

This review found that implementation scientists demonstrate a considerable interest in attitudes as a construct. However, investigators have not yet adopted a standard definition of attitudes. In addition, their methods for measuring attitudes fundamentally differ, and this appears to reflect conceptual ambiguity or inconsistency. As others have observed, implementation research often lacks standardization for the conceptualization and measurement of constructs. But remedies are immediately available [1, 36, 63]. Over several decades, psychology has developed standardized definitions, along with measurement approaches that are supported by strong psychometrics, including predictive validity [23].

This review also found that the authors rarely articulated how attitude data were analyzed, and most did not present any empirical results labeled as attitudes. Few explained why attitudes matter, either in and of themselves or in relation to other study variables. Consequently, there are many missed opportunities for investigators to apply validated causal models of behavior that can inform the analytic plan and justify decisions to test the relationship between attitudes and other variables. Psychology has also generated a large literature documenting the role of attitudes within causal models of behavior, revealing a parsimonious set of constructs that are proximally influencing and influenced by attitudes [23]. Investigators studying implementation of evidence-based practices can evaluate theorized relationships between attitudes and other constructs in these predictive models [1]. Such tests could inform the development and evaluation of effective implementation strategies.

## Data Availability

The dataset supporting the conclusions of this article is available from the Penn ALACRITY Data Sharing Committee by contacting the research coordinator, Kelly Zentgraf, at zentgraf@upenn.edu, 3535 Market Street, 3rd Floor, Philadelphia, PA 19107, in https://hosting.med.upenn.edu/cmh/people/kelly-zentgraf/
