# Correction to: Attitude theory and measurement in implementation science: a secondary review of empirical studies and opportunities for advancement

**DOI:** 10.1186/s13012-022-01204-9

**Published:** 2022-05-23

**Authors:** Jessica Fishman, Catherine Yang, David Mandell

**Affiliations:** Perelman School of Medicine, University of Pennsylvania, 3535 Market Street, Floor 3, Philadelphia, PA 19104 USA

**Correction to: Implement Sci 16, 87 (2021)**

**https://doi.org/10.1186/s13012-021-01153-9**

Following publication of the original article [1], it was reported that a number of references were numbered incorrectly or missing. The original article has been updated with the corrected references.
